# Use of a Real-Time Remote Monitoring Network (RTRM) to Characterize the Guadalquivir Estuary (Spain)

**DOI:** 10.3390/s120201398

**Published:** 2012-02-01

**Authors:** Gabriel Navarro, Isabel Emma Huertas, Eduardo Costas, Susana Flecha, Manuel Díez-Minguito, Isabel Caballero, Victoria López-Rodas, Laura Prieto, Javier Ruiz

**Affiliations:** 1 Departamento de Ecología y Gestión Costera, Instituto de Ciencias Marinas de Andalucía, ICMAN-CSIC, Puerto Real, 11519, Cadiz, Spain; E-Mails: emma.huertas@icman.csic.es (I.E.H.); susana.flecha@icman.csic.es (S.F.); isabel.caballero@icman.csic.es (I.C.); laura.prieto@icman.csic.es (L.P.); javier.ruiz@icman.csic.es (J.R.); 2 Departamento de Producción Animal, Facultad de Veterinaria, Universidad Complutense de Madrid, Madrid, 28040, Spain; E-Mails: ecostas@vet.ucm.es (E.C.); vlrodas@vet.ucm.es (V.L.-R.); 3 Grupo de Dinámica de Flujos Ambientales, Centro Andaluz de Medio Ambiente, Universidad de Granada, Avda. del Mediterráneo, Granada 18006, Spain; E-Mail: mdiezm@ugr.es

**Keywords:** real-time remote monitoring network, water quality node, Guadalquivir estuarine ecosystem, turbidity control

## Abstract

The temporal variability of hydrological variables in the Guadalquivir estuary was examined during three years through a real-time remote monitoring network (RTRM). The network was developed with the aim of studying the influence of hydrodynamical and hydrological features within the estuary on the functioning of the pelagic ecosystem. Completing this data-gathering network, monthly cruises were performed in order to measure biogeochemical variables that are indicative of the trophic status of the aquatic environment. The results showed that several sources of physical forcing, such as wind, tide-associated currents and river discharge were responsible for the spatio-temporal patterns of dissolved oxygen, salinity and turbidity in the estuary. The analysis was conducted under tidal and flood regime, which allowed us to identify river discharge as the main forcing agent of the hydrology inside the estuary. In particular, episodes of elevated turbidity detected by the network, together with episodes of low salinity and dissolved oxygen were closely related to the increase in water supply from a dam located upstream. The network installed provided accurate data that can be rapidly used for research or educational applications and by policy-makers or agencies in charge of the management of the coastal area.

## 1. Introduction

Aquatic ecosystems, including freshwater, estuarine and coastal environments are subjected to increasing anthropogenic pressure due to resource extraction (e.g., fisheries and water use), pollution, invasion of allochthonous species, and changes in the quantity and seasonality of hydrologic flows [1]. In addition, human activities have greatly accelerated the inputs of nutrients to estuaries, particularly over the past century, which has caused the increase in primary production and a widespread eutrophication [2]. The Guadalquivir estuary, located in the Southwestern Iberian Peninsula, has experienced profound modifications in relation to its original morphology and natural dynamics. During the last five decades, a substantial part of the forest and wetlands around the riverside have disappeared. As a result of these actions, the current shape of the estuary is characterized by a main channel surrounded by tidal creeks without any significant intertidal zones. The reduction of the tidal prism, along with other factors, has also altered the river mouth and the contour of the adjacent coastline of the Gulf of Cadiz [3]. At present, the estuary and its immediate surroundings host 1.7 million inhabitants, which are clustered in some 90 population settlements. Despite its socio-economic and environmental significance and its influence on the continental shelf of the Gulf, until very recently only a few studies had been carried out in the estuary. These works were mainly focused on fisheries, decapod crustacean population dynamics [4–6] and the monitoring of heavy metal concentrations after the Aznalcollar mining waste spill [7,8].

However, to date there was a clear lack of an integrated study that assessed the hydrodynamics of the estuary and the functioning of its pelagic ecosystem. With the aim of filling this gap, an interdisciplinary research program was initiated in the area in 2007, whose overall goal was to establish an integral method capable of diagnosing and forecasting the consequences of human activities on the Guadalquivir estuary dynamics. Part of the results generated in the framework of this initiative have been recently published [3,9] in order to provide insights into the tidal propagation and transformation within the estuary under the influence of different river flows regimes. The presence of different regimes modify the hydrodynamical and hydrological features of the estuary and river mouth, thereby affecting the ecosystem functioning [9,10]. The turbidity results from the presence of colored dissolved organic matter (CDOM; e.g., humic substances) and suspended particulate matter (SPM) in the water column. SPM may include clay and silt (e.g., total suspended sediment, TSS), and detritus and organisms (algae and zooplankton). Usual sources of turbidity in estuarine waters are numerous and varied, such as soil erosion from construction, forestry, or agricultural sites; waste discharge; urban runoff; eroding stream banks; stirred-up bottom sediments from flooding, dredging, boating and jet-skiing activities, or bottom-feeding animals; and excessive algal growth. In addition, natural runoff, water turbulence from storms, and wave action can also cause water turbidity. In turbid and nutrient-rich estuarine systems, light availability may be the most important factor controlling biomass-specific productivity [11,12]. In these systems, the restricted light availability may alter phytoplankton production in two ways: by regulating the maximum attainable biomass in the system [11] and by stimulating physiological acclimation to low light conditions [13]. It is well known that turbidity has an impact on light absorption by phytoplankton, which clearly restricts the concentration of dissolved oxygen in the water column. All these features brought about by the presence of suspended matter in the medium significantly alter the entire ecosystem functioning. An efficient management of the estuarine ecosystems requires the implementation and use of monitoring tools able to track changes in water quality and quantity in time. Such changes are best contextualized using synoptic data that help to identify the processes occurring throughout the entire riverine landscape [14]. Accordingly, a real-time remote monitoring (RTRM) network was deployed in the Guadalquivir estuary, which allowed us to measure the meteorological, hydrodynamical and hydrological patterns in the area during three years. Several other nodes were also distributed throughout the estuary to complete the information gathering [15]. The network provided data with a high degree of temporal resolution within an Eulerian framework, and permitted a constant surveillance to rapidly detect any changes and trends in critical physical and biogeochemical indicators [16]. Specifically, the network installed in the Guadalquivir estuary supplied detailed data suitable for research, policy-makers, government agencies and educational/outreach applications in charge the management of the nearby coastal fringe. In addition, the remote sensing analysis provided means to meet the mapping and monitoring requirements in the estuary. Optical sensors, such as Moderate Resolution Imaging Spectroradiometer (MODIS), have improved freshwater monitoring capabilities and, in turn, the capacity to assist aquatic ecosystems managers. Therefore, the aim of this study was to analyze the spatio-temporal variability of water quality parameters in the Guadalquivir estuary and the adjacent coastal area by using data obtained from a RTRM network along with the information collected by sampling cruises and satellite.

## 2. Study Area

**Figure 1.**
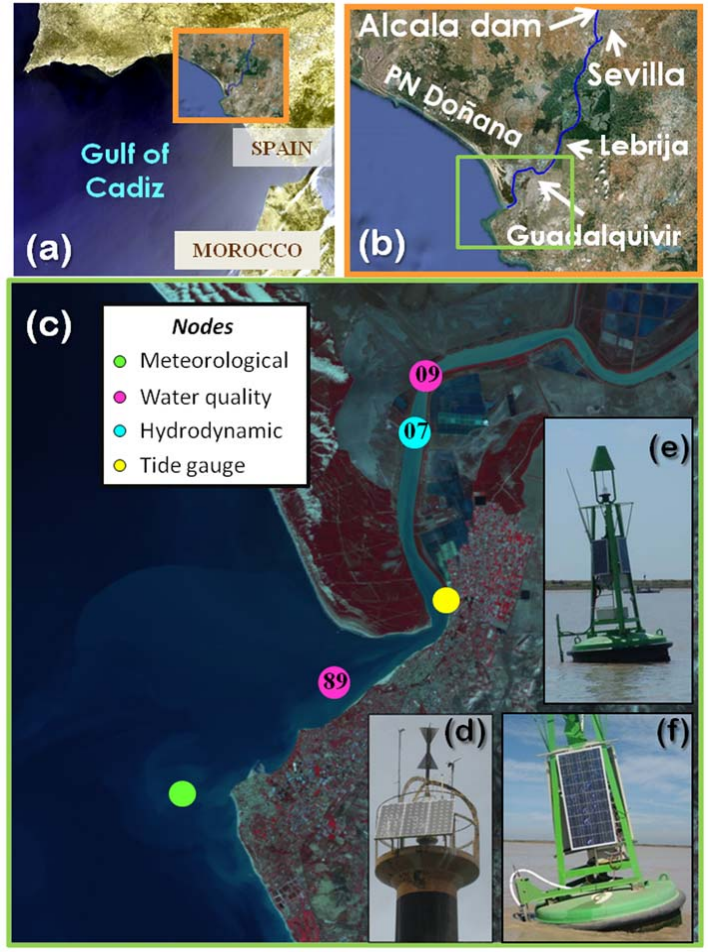
(**a**) Location of the Guadalquivir estuary in the SW Iberian Peninsula; (**b**) Zoom of the study area; (**c**) Locations of various stations of the RTRM (hydrodynamical, hydrological and meteorological nodes) and tide gauge station; (**d)** Photograph of the Salmedina buoy (meteorological node); (**e**) Photograph of the hydrodynamical node (node 07); (**f**) Photograph of the water quality node (node 09).

The Guadalquivir estuary is located on the SW coast of the Iberian Peninsula (Figure 1(a)). The source of the river is placed in the Cazorla mountains at about 1,400 m above sea level, and it flows into the Gulf of Cadiz; its total length is 680 km and it drains a basin of 63,822 km^2^ [17]. The estuary extends upstream of the Alcalá del Río dam, which is situated 110 km from the river mouth at Sanlucar de Barrameda (Figure 1(b)). The tidal influence extends up to the dam and the maximum tidal range at river mouth is 3.5 m during the spring tides [3]. This mesotidal estuary presents several notable characteristics, including the largely-protected estuary marshes forming part of the Doñana Natural and National Parks, which are considered a UNESCO-MAB Biosphere Reserve. The part of the river between Sanlucar de Barrameda and Seville is unique, as it is the only navigable river channel in Spain, which supports a relatively heavy commercial and tourist traffic. This activity requires maintenance of a minimum depth of 6.5 m in the channel, which involves an annual dredging of the course that is partially responsible for the estuary turbidity. Moreover, the estuary has undergone a rapid anthropogenic development, particularly in recent decades, for agricultural and fisheries purposes [18].

**Figure 2.**
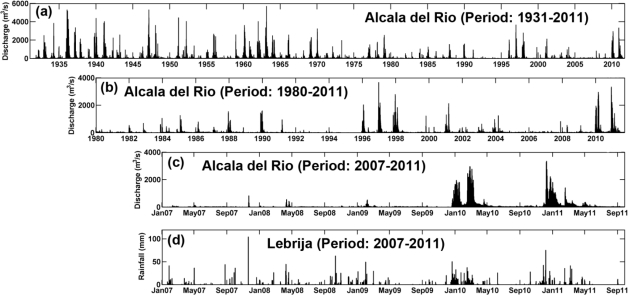
(**a,b,c**) Daily average river discharge (in m^3^/s) from the Alcalá del Río dam during different time periods; (**d**) Daily rainfall (in mm) measured by a meteorological station located in Lebrija from 2007 to 2011.

The freshwater discharges from the Alcalá del Río dam (Figure 1(b)) represent approximately 80% of the water supply received by the estuary (Figure 2). These discharges have a clear seasonal frequency and reflect the effects of the regulation of the hydrographic basin upstream of the dam. Freshwater contributions to the estuary have decreased by an average of 60%, from approximately 5,000 hm^3^/year in 1931–1981 to 2,000 hm^3^/year in 1981–2000, with a greater reduction during dry-year cycles (Figure 2). Recently, [3] have established two regimes depending on the hydrodynamics within the estuary. The most common regime is characterized by good weather conditions, low freshwater discharge (Q_d_ < 40 m^3^/s) and tidal dominance. These conditions represent the basic state of the estuary, which is slowly modulated by spring-neap cycles and by the local wind and wind waves. Under this regime, with N < 0.1 (where N stands for the Estuary Number), the estuary is tidally dominated. The river current velocity is within the range of 1–5 cm/s, which is much lower than maximum tidal velocities of 1 m/s and the river contribution to the water level can be considered negligible. Figure 2 illustrates that during the period of study the tidally-dominated regime was achieved between May and December of 2008, between March and December of 2009 and between May and November in 2010.

On the other hand, the fluvial-dominated regime is present when the river discharge exceeds 400 m^3^/s. Under these conditions, the estuary is no longer controlled by the tide and becomes fluvially-dominated. The fluvial current is equivalent or larger than the maximum tidal velocities [3], and much higher than the low-frequency, residual tidal current. Figure 2 shows that during April 2008, between January and February of 2009 and from January to April 2010, the river discharge was higher than 400 m^3^/s. Consequently, under this regime, N >> 0.1 surpassing the tidally-dominated regime [3].

## 3. Material and Methods

### 3.1. Real-Time Remote Monitoring Network and Data Collection

The real-time remote monitoring (RTRM) network comprised three different stations operated by the Instituto de Ciencias Marinas de Andalucía (ICMAN-CSIC) that provide meteorological, hydrographic and water quality information. The acquisition of remote data from continuous *in situ* monitoring offers important early warning information to decision-makers, which facilitates quick and adequate management responses. RTRM technologies have lately emerged as economically-viable means of recording key hydrological parameters [16]. The platform network allowed the collection of data with a high degree of temporal resolution within an Eulerian framework. The data management and control server (DMC), set up at the ICMAN-CSIC premises, was configured as a TCP-IP server that gathered raw data, status variables and communication events sent by the network nodes, which were stored in a database. As this database may contain sensitive data, it could only be accessed locally. However, after submitting the acquired raw data to a quality control, all of the scientific information was shared via a secondary FTP server and displayed the monthly times series of the data in real time through the project website (http://www.guadalquivir.csic.es). These figures have been created by MATLAB(c) software. The DMC server also allowed the operator to have remote access to the nodes and dynamically changed certain measuring parameters, control variables or even update the node firmware.

The stations were strategically positioned along the estuary and river mouth and maintained since 2008 (Figure 1). The meteorological station was installed in May 2008 on the Salmedina buoy located over the continental shelf off Chipiona (Figure 1(c,d)). The system contained an array of sensors for meteorological measurements (air temperature, relative humidity, incident solar radiation, barometric pressure and wind speed and direction, see details on Table 1), which were calibrated by the manufacturers. In particular, barometric pressure was obtained with a Young 61,202 L barometer, air temperature and relative humidity data were acquired via a Geonica STH-5031 instrument (Geonica©, Spain), wind parameters were measured using a marine wind sensor (R.M.YoungWind Monitor) and incident solar radiation was collected by a pyranometer (model Licor Li200 LiCor Biosciences, Lincoln, NE, USA). Although different measuring intervals could be selected, this station was designed to sample every second, and the average, maximum, and minimum values for a 10-min interval were sent to the DMC by telemetry. A Geonica Hydrodata 2008CP datalogger served as the central processing unit of the system and the energy supply came from a gel battery charged by a bank of three solar panels connected in parallel. Bi-directional communication between the meteorological station and the DMC enabled the instruments and sensors installed to be controlled and serviced remotely. To validate the quality of the meteorological data collected through the RTRM network, our records were contrasted with external data collected by another station belonging to IFAPA, part of the Junta de Andalucía (http://www.juntadeandalucia.es/agriculturaypesca/ifapa/ria/). Correlation between both records was very good for all the meteorological parameters [9].

The water dynamic node (Figure 1(c,e)) comprised three modules: power, measurement and telemetry. The power module consisted of a rechargeable 12-volt/50-ampere-hour gel battery housed in a polyester box, a 16-volt/1 O-ampere solar regulator and a bank of three 45-watt solar panels connected in parallel. The battery capacity supplied the system with enough autonomy to take two-minute profiles every five minutes for 10 days. In practice, taking into account the average insulation of the study area, this means an annual coverage higher than 99.9%. The measurement module was equipped with an acoustic Doppler current profiler (ADP, see details on Table 1) operating at a frequency of 1,000 kHz, with a cell size of 1 m, and with an integration period of 2 min and four profiles per hour. This ADP was used to measure both current speed and direction in the water column within one-meter cells below the navigation buoy. The Aquadopp ADP had to be programmed independently from the telemetry module, as both modules work asynchronously. The telemetry module included a Geónica S.A. Hydrodata 2008CP data logger equipped with a Global System for Mobile Communications/General Packet Radio Service (GSM/GPRS) modem and high-gain antenna. This data logger can handle up to 24 logical channels. In the current nodes, this allows the network to monitor the following variables in real time: battery level, head pressure and temperature, compass pitch and roll, ADP error/status and the three (east, north, up) water velocity components of the upper six cells. The rest of the information registered by the ADP (velocity of the other 15 cells, beam amplitudes, *etc.*) was stored in internal memory and recovered during routine maintenance operations, usually once every three months. The water dynamic node was configured as TCP-IP clients, which allows them to more easily handle the dynamic IP-address assignation from the network provider. The compass was calibrated following the manufacturer′s specifications whereas the ADP was calibrated by the manufacturers. Currents measured by the water dynamic station were validated with a different ADP (ADP-1,000 kHz SonTek) that integrates a GPS and bottom tracking. During a tidal cycle, water velocity profiles were measured near the buoy (node 07) using the same equipment configuration (cell size, integration, time, *etc.*). The results showed that the water velocity profiles recorded by the RTRM stations were measured correctly (data not shown). This node (node 07) is located in the inner estuary (Figure 1(c)).

**Figure 3.**
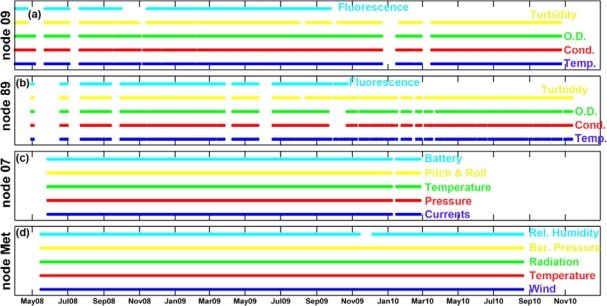
Bar chart showing daily data availability at the different water nodes. Panels (**a,b**) represent the water quality nodes (09 and 89 respectively). Colors indicate the measured parameters: blue, red, green, yellow and cyan denote temperature, conductivity, dissolved oxygen, turbidity, and chlorophyll fluorescence respectively. Panel (**c**) corresponds to data availability from the water dynamic station (node 07) whereas panel (**d**) shows data availability from the meteorological node (Salmedina). More than 97% the complete bulk of data that could have been recorded was accessible during the study period. Gaps in the panels indicate no data availability for a particular time period.

The two water quality stations were located in the inner part of the estuary (node 09) and in the river mouth (node 89) (Figure 1(c,f)). Each water quality node consisted of four modules: power, measurement, telemetry and hydraulics. The power module was a rechargable 12-volts/135-ampere-hour gel battery, a 16-volts/20-ampere solar regulator and a bank of three 120-watt solar panels connected in parallel. The battery capacity fuelled the system with enough autonomy to take 90-seconds, four depth profiles every 30 minutes for a total period of four days, which, taking into account the average insulation of the study area, permitted an annual coverage higher than 95%. The measurement module was equipped with a Seabird Ellectronics Inc (Bellevue, WA, USA) CTD-SBE16plus with external sensors to measure dissolved oxygen (Sea-Bird SBE-43), a Turner Designs (Sunnyvale, CA, USA) CYCLOPS-7 turbidimeter (hard-wired for ×l gain) and CYCLOPS-7 fluorometer (hard-wired for ×10 gain) (see details on Table 1). The hydraulic module was comprised by several parts: a polyester box with four SHURflo (Cypress, CA, USA) model number 8000-543-238 12-volt/48-watt suction pumps and a single flow meter (1 to 15 liters per minute), a 50-micron filter batch to avoid early degradation of the pumps and some silicon piping that permitted water sampling from different depths (one to four meters in this network). As in the water dynamics nodes, the telemetry module included a Hydrodata 2008CP data logger equipped with a GSM/GPRS modem and high-gain antenna. Nevertheless, this data logger had been adapted to run the main control loop of the system as well as the data logging and telemetry tasks. More information regarding the control loop is given in [15]. The temperature, conductivity and dissolved oxygen sensors were calibrated by manufacturers, whereas turbidimeter and fluorometer were calibrated in the lab [9]. The standard measuring rate was set up at two cycles per hour at 1 meter depth. In order to validate the quality of the data collected by the water quality nodes, data from monthly cruises and those carried out by the Agencia Andaluza del Agua were considered (next section). A comparison between the water quality nodes and *in situ* measurements showed a good validation for all parameters [9]. Figure 3 confirms that data availability was quite good since more than 97% of the total expected data could be acquired.

### 3.2. Cruise Sampling and Multiple Data to Complete the RTRM Data

Monthly oceanographic cruises started in June 2007 and were maintained until August 2009. In each cruise, surface water samples were collected at several stations along the estuary with Niskin bottles with the aim of measuring nutrients, chlorophyll-a (CHL) and total suspended solids (TSS). At each sampling site, a CTD cast was performed with a SBE19 equipped with external sensors (dissolved oxygen, SBE43; turbidity, Turner Designs Cyclops-7; fluorometer, Turner Designs Cyclops-7). Water samples measurements and CTD data were used to check the RTRM water quality information and to contrast that of other important physical and biogeochemical variables [9].

Nutrients (nitrate, nitrite, phosphate and silicate) were analyzed with a TRAAC800 autoanalyser following the techniques of [19]. Two replicates of filtered water (12 mL, Whatman GF/F filters) were taken at each station (nodes 09 and 89) and stored at −20 °C until analysis in the laboratory. The standard deviation of each replicate was under 0.06 and 0.01 μM for nitrate and phosphate, respectively. Chlorophyll analysis was conducted by filtering samples through Whatman GF/F glass fiber filters (0.7 mm pore size), extracting in 90% acetone, and measuring chlorophyll-a [CHL] by standard fluorometric methods following JGOFS protocols [20] using a Turner Designs Model10. The fluorometer was calibrated using pure chlorophyll a from the cyanobacterium *Anacystis nidulans* (Sigma Chemical Co.) with the concentration being determined spectrophotometrically. Total concentrations of suspended particulate matter were measured gravimetrically on preweighted Whatman GF/F filters according to the JGOFS protocols [20].

The diffuse attenuation coefficient for photosyntetically active radiation (PAR) was calculated by the following expression [21]:

(1) $$K_{d}\left(\text{PAR}\right)=K_{W}+K_{C}\;[\text{CHL}]+K_{S}\;[\text{TSS}]$$

where K_W_ (0.26 m^−1^) is the attenuation due to water alone, and K_C_ (0.017, (mg·chl·m^−3^)^−1^m^−1^) and K_S_ (0.074, (mg·L^−1^)^−1^m^−1^) stand for the specific-attenuation coefficient of chlorophyll and total suspended solid, respectively. The diffuse attenuation coefficient (K_d_) was used to estimated the lower limit of the euphotic zone (Z_1%_) defined as the depth where the PAR represents 1% of the surface radiation, following the Lambert-Beer law [22]:

(2) $$Z_{1\%}=\frac{\text{Ln}\;(0.01)}{K_{d}}$$

In addition, daily discharge data from the Alcalá del Río dam was obtained from the Regional Water Management Agency (Agencia Andaluza del Agua, Junta de Andalucía) whereas daily rainfall measurements were acquired by a meteorological station located in Lebrija (Figure 1(b)). The water level data was recorded with tidal gauges property of Puertos del Estado placed in the port of Bonanza (Figure 1(c)).

### 3.3. Remote Sensing Data

RGB MODIS images were downloaded from the National Aeronautics and Space Administration (NASA) server (http://lance-modis.eosdis.nasa.gov). Images were processed and projected using MATLAB^©^ software. Satellite imagery can resolve patterns of turbidity on a large spatial scale but is confined to surface data. The turbid plume was detected by the backscattering characteristics of the surface waters in the vicinity of the mouth area and could be distinguished from the surrounding waters by its high optical reflectivity properties.

## 4. Results

### 4.1. General Patterns

Figure 2 displays the daily river discharge at Alcalá del Río dam since 1931. It is evident that, the discharge has decreased with time, particularly over the last four decades. It can be also observed that increases in river discharge are associated to the rise in the local rainfall (Figure 2(d)). This trend is more obvious since 2007 and during the period of study. These events normally coincide with the winter and spring months (November 2007, April 2008, February 2009, January to April 2010 and January to March of 2011, Figure 2(c)). It has been recently demonstrated that the river discharge can be considered one of the main forcing agents of the hydrodynamics [3] and hydrology [9] of the estuary.

**Figure 4.**
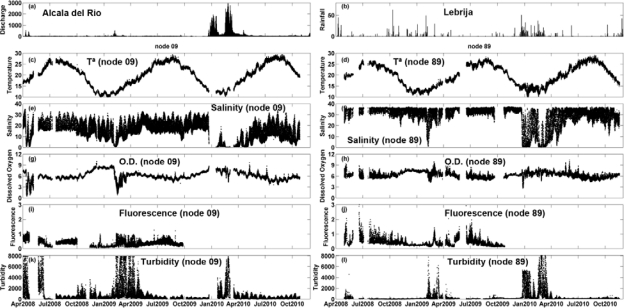
(**a**) Daily average river discharge (in m^3^/s) from the Alcalá del Río dam from April 2008 to October 2010; (**b**) Daily rainfall (in mm) measured at the meteorological station located in Lebrija during the same period; (**c** to **l**) Real-time remote monitoring data observation at node 09 (left panels) and node 89 (right panels). Temperature records (in °C) are indicated in panels **c** and **d**, salinity in **e** and **f**, dissolved oxygen (in mg/L) in **g** and **h**, fluorescence (in relative units) in **i** and **j** and turbidity (in FNU) in **k** and **l**.

The high-resolution, long term hydrological measurements obtained in the inner station (node 09) and in the river mouth (node 89) are shown in Figure 4. Data reveal wide fluctuations of the water quality variables during the study period that lasted more than two years. Temperature ranged between 9.6 °C (in January–February) and 29.4 °C (in July–August) and showed an obvious seasonal pattern characteristic of temperate latitudes, with maxima in summer and minima in winter. The temperature in the river mouth (node 89, Figure 4(d)) was lower during summer and higher in winter in relation to those detected in the inner station (node 09, Figure 4(c)), where temperature also exhibited a high variability (Table 2).

Salinity oscillated from near 0.07 during high discharge periods to more than 37 in the river mouth whereas the maximum value in the inner node was lower than 35. Moreover, salinity in this node was always lower than in the estuary mouth node (*t*-student, *P* < 0.01). As the salinity is markedly influenced by the runoff water, the lowest salinity was consequently registered during the rainfall season (Figure 4(e)). In contrast, during the dry season (*i.e.*, from August to November 2007) salinity kept values around 15 (±7) and 30(±9) in the inner station and in the estuary mouth, respectively.

**Figure 5.**
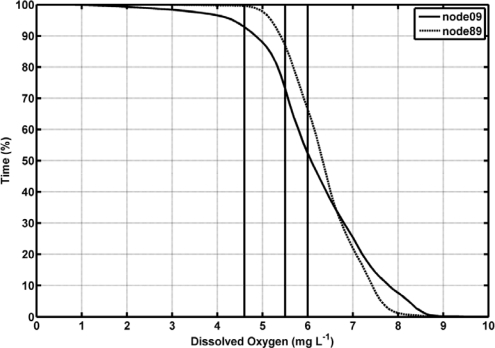
Percentage of time in which dissolved oxygen (DO) remained at the measured concentrations in nodes 09 (solid line) and 89 (dashed line). Vertical lines indicate DO concentrations of 4.59, 5.5 and 6 mg/L, which mark the presence of hypoxia in the water column according to different sources (more details are given in the text).

**Figure 6.**
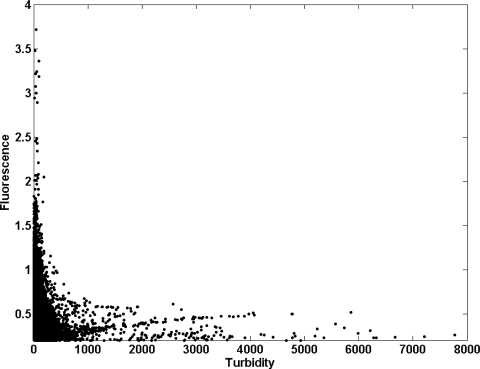
Turbidity (in F.N.U.) *vs.* fluorescence (in r.u.) measured by RTRM in the node 89.

Dissolved oxygen (DO) ranged from 0.52 mg/L, with the minimum being achieved in April 2008, to 10 mg/L in the inner station (node 09, Figure 4(g)), whereas in the estuary mouth this variable varied from 2.66 to 10.56 mg/L (node 89, Figure 4(h)). DO was always higher in the river mouth than in the inner node (t-student, *P* < 0.01). The average value of DO and standard deviation (SD) of the whole temporal time series were 6.10 ± 1.23 in the inner estuary node and 6.37 ± 0.78 in the node located at the estuary mouth (Table 2). According to the United States Environmental Protection Agency (EPA), these values may be indicative of the presence of a severe hypoxia in the system. In addition, DO showed a negative correlation with temperature in both nodes (−0.60 and −0.69 for nodes 09 and 89, respectively; Table 3). In aquatic systems, oxygenation is the result of an imbalance between the process of photosynthesis, degradation of organic matter, re-aeration [23], and physicochemical properties of the water [24]. Recently, [25] established 4.59 mg/L as the median lethal oxygen concentration (LC50, for 90% of the experiments conducted) and the Canadian Ministry of Environment recommends DO concentration in temperate rivers to be maintained at levels higher than 6.5 mg/L for the proper development of fish and higher than 6 mg/L for the preservation of the aquatic biota in general [26]. Therefore, the DO levels measured in both nodes indicated that the proliferation of aquatic biota may be constraint within the estuary (Figure 4). Figure 5 shows the percentage of time in which DO concentration remained below these reference levels in the inner and river mouth nodes established in the Guadalquivir estuary. In the former, DO concentration was lower than 4.9 mg/L during 5% of the study period whereas in the estuary mouth node such a low concentration was hardly achieved. On the other hand, DO was always lower than 6 mg/L (optimum for the preservation of the aquatic biota) during 50% and 40% of the time in the inner and estuary mouth nodes respectively. In addition, the turbidity pattern showed a patent variability, with maxima being observed during high runoff and minima coinciding with decreases in the river discharge. The RTRM allows us to analyze the control that turbidity exerts on the primary production in the estuary mouth. Figure 6 shows that high fluorescence values in this part of the estuary were exclusively reached when turbidity kept low values. In turbid environments, light availability plays a fundamental role in regulating primary productivity as it is the energy source for phytoplankton growth [27–29]. It has been also documented that the illumination conditions in the water column affect the taxonomic structure of the phytoplankton community and inter-specific algal competition [30–32].

**Figure 7.**
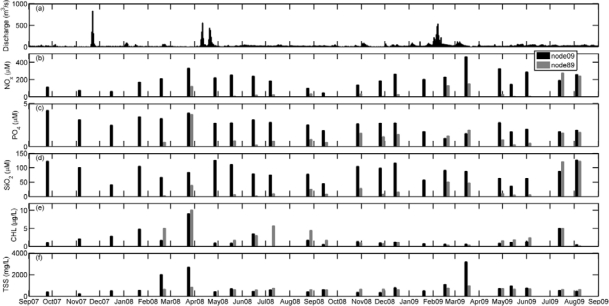
(**a**) Daily average river discharge (in m^3^/s) from the Alcalá del Río dam during two years. Temporal patterns of (**b**) NO_x_ (μM), (**c**) PO_4_ (μM), **(d**) SiO_2_ (μM), (**e**) chlorophyll (μg/L) and (**f**) total suspended solids (TSS, mg/L) measured in node 09 (black bar) and node 89 (grey bar). Data were obtained in monthly cruises performed from September 2007 to September 2009.

Nevertheless, phytoplankton development in aquatic systems also depends on the availability of nutrients. In the Guadalquivir estuary, the concentration of nitrate plus nitrite (NO_x_) was characterized by high values in relation to those reported in other estuarine areas located in the Atlantic side of the Iberian Peninsula, such as the Tagus river [33] and the Guadiana river [34]. In particular, in the inner station NO_x_ levels exceeded those found in the estuary mouth node (Figure 7, *t*-student *P* < 0.01). Even though NO_x_ concentration exhibited a clear seasonal variation, the values measured ranged from 49 to 461 μM at node 09 and from 1.89 to 278 μM at node 89 (Table 2), which suggests that nitrogen was not a limiting nutrient for phytoplankton growth in this area. Nitrate was the major nitrogen source throughout the year (data not shown). In addition, NO_x_ maxima were obtained under periods of high river discharge and, in fact, significant negative correlations were found between salinity and NO_x_ (r = −0.42; *p* < 0.05, Table 3) and between silicate and salinity (r = −0.80, p < 0.05, Table 3) in the node 09. The seasonal pattern of phosphate concentration was similar than that of silicate, with maximum values of both nutrients being registered during winter and spring (coinciding with the maxima in river discharge, Figure 7). At both stations, the SiO_2_:PO_4_ ratio was higher than 3 and silicate concentrations lower than 5 were barely found, which indicates that silicate was not a limiting nutrient [33]. Although the SiO_2_:PO_4_ ratio remained always above 3, the lowest PO_4_ concentration measured was 0.21 μM, which suggests that the phytoplankton growth was not limited by phosphate either (phytoplankton semisaturation constants for nitrate and phosphate are 0.5 μM [35] and 0.05 μM [36], respectively). Furthermore, the concentrations of nutrients found in the river mouth were higher than those previously measured in the adjacent continental shelf [10] and in the open ocean area of the Gulf of Cadiz [37]. Higher values were even detected in the inner estuary (Table 2, Figure 7).

**Figure 8.**
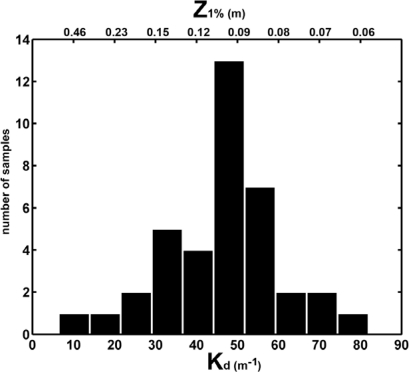
Histogram of K_d_ and photic depth (Z_1%_) for node 09 and node 89.

These elevated nutrients levels should allow the maintenance of a high phytoplankton biomass and particularly considering the shallowness of the estuary. However, the chlorophyll concentration measured in the inner estuary and the estuary mouth stations (Figure 7(e)) showed varying values that ranged from 0.2 to 10 μg/L (Table 2), with a strong seasonal variation. Chlorophyll was also lower than that reported in the continental shelf of the Gulf of Cadiz [10] and a statistical relationship between nutrients and chlorophyll in node 89 could not be found. This finding reinforces the statement that a strong light-limitation due to the high turbidity and TSS concentration was present in the Guadalquivir estuary (Figure 6). Mean values of TSS (844 mg/L and 698 mg/L for nodes 09 and 89 respectively, Table 2) are considerably higher than others measured in important estuaries such as the Amazonas (200 mg/L), Mississippi (362 mg/L), Orinoco (136 mg/L), Danube (325 mg/L) and Niger (208 mg/L). These high TSS values along with chlorophyll concentration, caused the elevated values of the attenuation coefficient (K_d_) for the PAR obtained (Figure 8), which indicates that primary production was light limited in the water column. Figure 8 displays the histogram of the K_d_ values in both nodes. With these K_d_ values, the photic depth (Z_1%_) was always less than 1 m depth, well above the usual Z_1%_ in aquatic environments. This demonstrates that PAR poorly penetrated into the water column. It is worth to mention that primary productivity in estuaries can be potentially higher than in nearby coastal areas although, due to light limitation, this potential is seldom reached [38], as it is the case of the Guadalquivir estuary.

### 4.2. Fluvially-Dominated Regime

The general pattern of hydrological and biogeochemical variables found in the Guadalquivir estuary allows us to conclude that river discharge is one of the main forcing agents of the biological activity in the estuary. In fact, the spatio-temporal variability of TSS, turbidity and nutrients (Figure 7) was clearly connected to the water supply from the dam.

**Figure 9.**
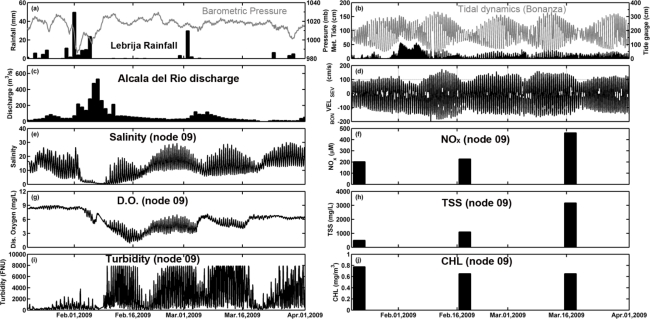
(**a**) Daily rainfall (black bar) registered by the meteorological station located in Lebrija and atmospheric pressure (grey line) recorded at the meteorological node; (**b**) Meteorological gauge (black bar) and tide gauge (grey line) measured at Bonanza; (**c**) Daily average river discharge from the Alcalá del Río dam; (**d**) Water current velocity measured by the hydrodynamic node at 1-m depth (negative, downstream current in the estuary). Real-time remote monitoring data observation at node 09 of salinity (**e**), dissolved oxygen (**g**) and turbidity (**i**). Temporal distributions of NO_x_, total suspended solids and chlorophyll distribution at node 09 (**f, h** and **j**, respectively).

When the river discharge exceeds 400 m^3^/s, the estuary becomes fluvially-dominated and the fluvial currents are of the same magnitude or larger than the maximum tidal velocities, and much higher than the low-frequency, residual tidal currents [3]. As depicted in Figure 2, the increase in river discharge was strongly linked to the rise of local rainfall. The number of storms and therefore the annual rainfall on the Guadalquivir watershed produced by these events show a periodicity of 12 years [39]. Freshwater discharges from the Alcalá del Río dam, regardless of precipitation, also occur on a synoptic scale during the passage of Atlantic storms [3]. The coincidence between the augment in rainfall and the decrease in barometric pressure related with Atlantic storms has been plotted in Figure 9(a).

**Figure 10.**
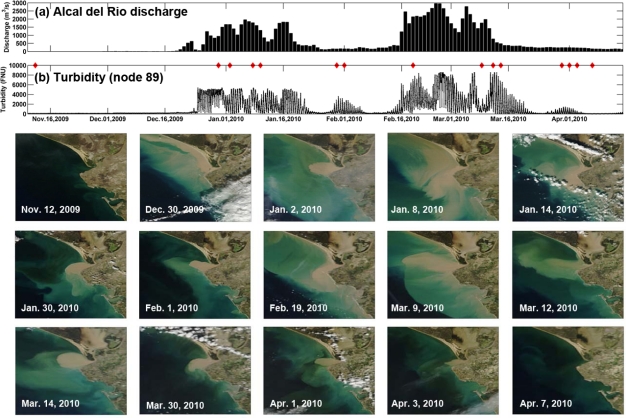
(**a**) Daily average river discharge (in m^3^/s) from the Alcalá del Río dam from November 2009 to May 2010; (**b**) Temporal distribution of turbidity (in FNU) measured at node 89 during the same period. The rest of figures are RGB MODIS images captured in different dates (denoted by red diamonds) during the progression of the river plume from the river mouth to the adjacent continental shelf.

It can be observed that during the rainy season (Figure 9(a)), the salinity decreases drastically (Figure 9(e)) and the DO concentration falls under the reference level of 4.59 mg/L that is indicative of hypoxia in the estuary (Figure 9(g)). Therefore these climate-related events have a marked impact on the functioning of the aquatic ecosystem. For instance, during a high rainfall episode occurring at the beginning of February 2009, the discharge modified the tidal regime in the estuary (Figure 9(b)) and salinity values remained close to 0 (freshwater) during several days. Changes in the river discharge also affected the inputs of nutrients (NO_x_, Figure 9(f)) and total suspended matter concentration (TSS, Figure 9(h)), which considerably rose in the water column. These increments had a contrary effect on chlorophyll distribution and primary production became limited by the decrease in light availability due to the high presence of TSS. This feature conditions the dynamics of the entire estuary mouth and even farther, as the turbidity plume reached several hundred km^2^. This can be observed in Figure 10 where the development of a turbidity plume associated to a high river discharge event that occurred at the end of 2009 and beginning of 2010 was daily followed.

### 4.3. Tidally-Dominated Regime

On the other hand, during the dry season characterized by river discharges lower than 100 m^3^/s, the dynamics in the estuary mouth are dominated by tidal regime [3]. During this period, a standard spectral and harmonic analysis of the water quality parameters was performed in order to study the variability of the hydrological and biogeochemical variables in relation to tidal forcing [40]. Specifically, the analysis period spans from mid May to mid September 2010 for temperature, salinity, dissolved oxygen and turbidity and from June to September 2009 in the case of fluorescence (Table 4).

**Figure 11.**
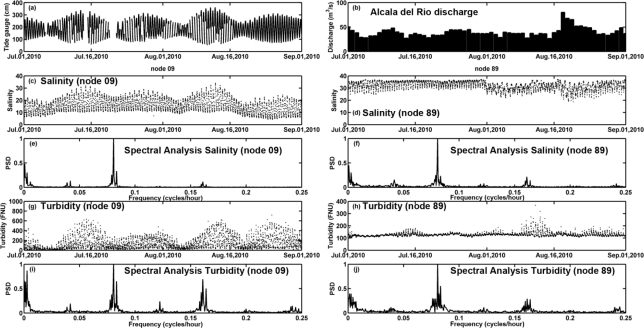
(**a**) Tide gauge; (**b**) Daily average river discharge from the Alcalá del Río dam. Temporal patterns of salinity at node 09 (**c**) and node 89 (**d**). Normalized power spectrum for salinity at node 09 (**e**) and node 89 (**f**). Temporal patterns of turbidity at node 09 (**g**) and node 89 (**h**). Normalized power spectrum for turbidity at node 09 (**i**) and node 89 (**j**).

It was found that the harmonic analysis explains a higher tidal variability in the inner estuary than in the river mouth (Table 4). Particularly, in the inner node, salinity and turbidity patterns were more related with tidal dynamics than the rest of parameters, as indicated by the values of the explained variance. In contrast, the temperature pattern was barely connected to the tidal regime, as this variable is mainly governed by the incident radiation. In fact, the main constituents in the temperature analysis were related with monthly periodicity (Table 4). For salinity and turbidity, the constituents associated with semidiurnal signals (M2 and S2 group) were the most energetic (Table 4) and represented more than 50% and 33% of the harmonic signals for salinity and turbidity respectively. Consequently, for both variables, the diurnal constituents (K1 and O1 groups) were less representative than the semidiurnal constituents. The quarter-diurnal overtides (M4 group) were mainly associated with turbidity and fluorescence distribution, which was especially generated in the inner node. These constituents of the tidal signal significantly contribute to the tidal asymmetry and the transport and accumulation of sediments [3]. Sixth-diurnal overtides (M6 group) represented less than 6% of the amplitude for each parameter. The amplitudes of the monthly and fortnightly constituents (MM and MSF respectively) also explained a high proportion of the variability, being higher in the inner node (09) than in the river mouth (node 89). The spring-tide periods of enhanced vertical mixing and enhanced turbidity that take place in muddy, strongly tidal systems lead to an overall decrease in the annually averaged chlorophyll concentration as compared to microtidal estuaries [41]. The relevance of spring and neap tide cycles on tidal dynamics is particularly important in the first stretch of the estuary, where the tidal range is reduced in comparison to its range in the open sea. This reduction was more significant under spring tides (40%) than under neap tides (10%). Figure 11 displays the harmonic analysis of turbidity and salinity related with tidal gauge and low river discharge.

## 5. Discussion

The Guadalquivir estuary plays a relevant role in the regulation of the pelagic ecosystem of the continental shelf of the Gulf of Cadiz and hence in the maintenance of the natural resources of the basin. The final stretch of the Guadalquivir river is a major driver of ocean circulation of the Atlantic coastal margin [42], also acting as a fertilizer through the pumping of permanent inorganic nutrients that are injected into the basin [10]. The continuous input of nutrients of a fluvial origin to the system favors the maintenance of high rates of primary production that are transferred to higher trophic components, including pelagic fish [10,18]. In fact, the ecological relevance of the area comprised between the Guadalquivir mouth and the coastal fringe of Doñana National Park was recognized through its declaration as a Fisheries Reserve.

The significance of the estuary on the trophic status and biological productivity of the continental margin of the Gulf of Cadiz is comparable to that of other river systems as significant as the Amazonas, Mississippi and Pearl, which govern the functioning of large basins such as the tropical Atlantic, the Gulf of Mexico and the South China Sea, respectively. In the case of the Amazonas River, whose mouth forms one of the world's largest estuarine areas with a plume extending 200 miles offshore, it was found that the discharge of inorganic nutrients represents an essential contribution to the biogeochemical inventory of the entire Brazilian continental shelf and its adjacent slope [43]. If we consider that the Guadalquivir estuary carries nitrate and phosphate concentrations that are up to two orders of magnitude higher than those measured at the Amazonas mouth [43], one order than those found in the Mississippi [44], and comparable to those reported in the Thames estuary [45] and the Seine [46], its impact on the regional biogeochemistry is evident.

However, the elevated concentration of nutrients measured in the Guadalquivir estuary was always higher than those considered limiting for phytoplankton growth [35,36]. The non-limitation by nutrients is furthermore confirmed by the lack of significant relationship between nutrients and chlorophyll concentration (Table 3). Another additional finding that supports the statement that in this nutrient-replete estuary light availability is the key factor that controls primary production, is the fact that nutrient concentration and TSS were higher in the inner node than in estuary mouth station, in opposition with the chlorophyll concentration that was higher in the estuary mouth. Therefore, according to the analysis conducted, it can be concluded that there is a strong light limitation for phytoplankton growth and productivity in the Guadalquivir estuary due to the presence of high turbid waters. In fact, when turbidity diminishes in the river mouth, fluorescence increases exponentially (Figure 6). This response coincides with that found in other turbid, nutrient rich estuaries where phytoplankton primary production is directly proportional to light availability [47], which in turn is controlled by turbidity [12]. Light-limited phytoplankton growth can occur consistently throughout the whole year [48] or episodically during specific seasons or periods [49,50]. In the Guadalquivir river estuary, this circumstance seems to be an annual feature.

This biological response is primarily controlled by seasonal changes in river discharge, winds associated with meteorological events and tides, as previously indicated [3,9]. The broad seasonal range of these forcing agents, generates strong patterns in the distributions of biogeochemical variables. The seasonal shift in hydrology from river-dominated (winter seasons) to tidally dominated flow is therefore, the principal factor controlling the patterns of nutrients and suspended matter in the estuary, which also constrains primary production rates. As many estuarine systems, the seasonal patterns of nutrient concentration and suspended matter in the Guadalquivir estuary were strongly linked to the freshwater inputs from a reservoir (in this case from the Alcala del Rio dam). This relationship has been studied in other Atlantic estuaries, such as the Tagus [33,51,52] and Guadiana [34,53].

The deployment of the RTRM has allowed to monitor the spatio-temporal variability of key hydrological parameters in the Guadalquivir estuary. The network was particularly useful because estuarine ecosystems are characterized by a high inherent variability [54]. RTRM permitted the collection of measurements of different hydrological, hydrodynamical and meteorological variables with a high temporal resolution and also a rapid access to researches to site-specific data (e.g., dissolved oxygen, turbidity, salinity) in the estuary. The elevated data availability and the good quality of the data obtained made the RTRM a powerful tool for monitoring and analyzing all the diverse processes that govern this kind of environments. Thus, in this markedly variable estuarine system, RTRM enabled the rapid implementation of an adaptive sampling that helped to characterize the aquatic ecosystem. Moreover, another advantage of the RTRM network is that it was shown to be capable of operating continuously in a reliable way during exceptionally hydrographic conditions, like a massive river discharges, high current velocities, and elevated water turbidity. Under these conditions, it is when the aquatic ecosystem becomes normally more altered by external circumstances that lead to the appearance of severe states, such as hypoxia, light limitation *etc.*

## 6. Conclusions

In conclusion, the development and establishment of the RTRM in the Guadalquivir estuary had a double benefit: (i) on the one hand, the data collected by the network could be used to understand the hydrodynamics and hydrological features governing the functioning of the aquatic ecosystem; (ii) on the other hand, real-time remote monitoring can be applied to generate early warnings for navigation, turbidity increases, hypoxia events, and toxic algal blooms. The data can also be sent automatically to any environmental protection agency and therefore, this type of network can be considered a powerful tool in an earlier decision making process to prevent or mitigate undesirable events.

## Figures and Tables

**Table 1.** Sensors equipment installed in different nodes.

**Node**	**Parameter**	**Characteristics**	**Model, Manufacturers**
**Meteorological**	Wind speed and direction	Range: <100 m/s, accuracy: 1 m/s	Wind Monitor, R.M. Young
	Air temperature	T range: −30 to 70 °C, accuracy: ±0.1 °C	STH-5031, Geonica
	Relative humidity	H range: 0 to 100%, accuracy: ±3%	
	Baromeric pressure	Range: 600–100 hPa, accuracy: ±0.5 hPa	61,202 L, Young
	Incident solar radiation	Range: 400 to 1,100 nm, error: <5%	Pyranometer Li200, Licor

**Hydrodynamical (node 07)**	Current profiler (ADP)	Maximum profiling range: 20 m	Aquadopp Profiler (1,000 kHz), Nortek
		Cell size: 0.3–4 m	
		Minimum blanking: 0.20 m	
		Velocity range: ±10 m/s	
		Accuracy: 1% of measured value	

**Water quality (nodes 09 / 89)**	Temperature	Range: −5 to 35 °C, accuracy: 0.0001 °C	SBE16+, Seabird Electronics Inc.
	Conductivity	Range: 0 to 9 S/m, accuracy: 0.0001 S/m	SBE16+, Seabird Electronics Inc.
	Dissolved oxygen	Range: 120% saturation, accuracy: 2% sat.	SBE43, Seabird Electronics Inc.
	Fluorescence	Range:0–500 μM/L, accuracy: 0.03 μM/L	Fluorometer Cyclops-7, Turner Designs
	Turbidity	Range: 0–3,000 NTU, accuracy: 0.04 NTU	Turbidimeter Cyclops-7, Turner Designs

**Table 2.** Descriptive statistics of the data collected in nodes 09 and 89 from April 2008 to October 2010.

	
	**Node**	**Mean**	**SD**	**CV**	**Min**	**Max**	**N**
RTRM data							

Temperature (°C)	node 09	19.98	5.24	0.26	9.63	29.37	38,297
	node 89	19.66	4.86	0.25	9.79	29.01	35,263
Salinity	node 09	15.24	7.87	0.51	0.07	34.98	38,297
	node 89	29.82	8.96	0.30	0.20	37.35	33,887
Dissolved Ox. (mg/L)	node 09	6.10	1.23	0.20	0.52	10.08	38,297
	node 89	6.37	0.78	0.12	2.66	10.56	33,875
Fluorescence (u.r.)	node 09	0.25	0.24	0.96	0	1.27	27,066
	node 89	0.27	0.28	1.01	0	3.72	27,260
Turbidity (FNU)	node 09	696.56	1,377.11	1.98	0	7,906.69	35,814
	node 89	338.35	934.84	2.76	0	8,494.17	34,980

Cruise data							

NO_x_ (μM)	node 09	199.36	97.35	0.49	49.62	461.22	24
	node 89	70.85	88.88	1.25	1.89	278.08	17
PO_4_ (μM)	node 09	2.54	0.76	0.30	0.95	4.19	24
	node 89	1.12	0.86	0.77	0.21	3.73	17
SiO_2_ (μM)	node 09	86.35	26.91	0.31	36.12	126.10	24
	node 89	29.86	37.67	1.26	2.14	122.33	17
CHL (μg/L)	node 09	2.09	2.04	0.98	0.52	9.05	24
	node 89	2.73	2.58	0.95	0.22	10.06	17
TSS (mg/L)	node 09	844.91	753.60	0.89	247.0	3172.0	24
	node 89	698.32	96.13	0.13	602.0	976	17

**Table 3.** Correlation coefficients (r) between water quality parameters and biogeochemical variables. Values marked with asterisk or cross are statically significant at *P* < 0.05 and *P* < 0.01 respectively. The rest of values are not significant.

**Node 09 Node 89**	**Sal.**	**D.O. (mg/L)**	**Fluor. (r.u)**	**Turbid. (FNU)**	**NO_x_ (μM)**	**PO_4_ (μM)**	**SiO_2_ (μM)**	**CHL (μM/L)**	**TSS (mg/L)**
Temp. (°C)	0.25 ^+^	−0.60 ^+^	0.01	−0.37 ^+^	−0.22	0.003	0.14	0.03	−0.28
	0.40 ^+^	−0.69 ^+^	0.34 ^+^	−0.22 ^+^	0.26	−0.30	0.29	0.18	−0.37
Salinity		−0.09 ^+^	0.09	−0.05 ^+^	−0.42 *	−0.15	−0.80 *	0.08	−0.14
		−0.03 ^+^	0.25 ^+^	−0.47 ^+^	−0.39	−0.53 *	−0.29	0.16	−0.78 ^+^
D.O. (mg/L)			−0.21 ^+^	−0.004	0.39 *	−0.06	−0.04	−0.05	0.18
			0.05 ^+^	−0.02 ^+^	0.03	0.11	0.11	0.05	−0.06
Fluor. (r.u)				0.60 ^+^	0.16	0.35	−0.04	0.45 *	0.51 ^+^
				−0.10 ^+^	−0.1	−0.31	−0.14	0.09	−0.31
Turbidity (FNU)					0.67 ^+^	−0.21	0.13	−0.21	0.56 ^+^
					0.25	0.24	0.12	−0.24	0.76 ^+^
NO_x_ (μM)						−0.13	0.12	0.02	0.67^+^
						0.54 *	0.97 ^+^	0.04	^+^ 0.22
PO_4_ (μM)							0.31	0.43 *	−0.04
							0.47 *	0.40	0.54 *
SiO_2_ (μM)								0.01	−0.14
								−0.01	0.09
CHL (μM/L)									0.29
									0.14

**Table 4.** Results of the harmonic analysis. VAR stands for the variance explained by the harmonic analysis. The amplitude for the different harmonics for each parameter is also indicated.

**Harmonics (amplitude)**												
	node	VAR	MM	MSF	K_1_	O_1_	M_2_	S_2_	M_4_	MS_4_	M_6_	2MS_6_
Temp.	09	8.7%	0.84	0.27	0.36	0.03	0.17	0.11	0.04	0.02	0.01	0.01
	89	5.9%	0.63	0.17	0.25	0.04	0.29	0.12	0.03	0.02	0.02	0.02
Salinity	09	70.7%	0.56	1.20	0.73	0.75	6.35	1.70	0.81	0.35	0.04	0.06
	89	61.9%	0.41	0.56	0.56	0.30	3.45	0.59	0.83	0.48	0.21	0.09
DO	09	21.8%	0.25	0.36	0.12	0.01	0.07	0.02	0.04	0.05	0.02	0.03
	89	25.8%	0.08	0.14	0.16	0.07	0.39	0.09	0.05	0.04	0.02	0.03
Fluor.	09	24.1%	0.02	0.16	0.04	0.01	0.05	0.01	0.04	0.03	0.01	0.01
	89	20.3%	0.04	0.07	0.05	0.01	0.07	0.01	0.02	0.01	0.01	0.005
Turb.	09	70.0%	56.4	95.2	10.7	10.1	107	51.7	80.1	49.3	12.4	8.70
	89	45.9%	4.35	8.55	0.50	1.96	13.7	7.53	4.59	2.51	1.26	1.29

**Variability explained (%) for each groups of harmonics**							

	**node**	**Monthly**	**Fortnightly**	**Diurnal**	**Semidiurnal**	**Quarter-diurnal Overtides**	**Sixth-diurnal Overtides**
Temp.	node 09	45%	14%	21%	15%	3%	1%
	node 89	40%	10%	18%	26%	3%	3%
Salinity	node 09	4%	10%	12%	64%	9%	1%
	node 89	5%	8%	11%	54%	18%	4%
DO	node 09	26%	37%	13%	10%	9%	5%
	node 89	8%	13%	21%	45%	8%	5%
Fluor.	node 09	5%	42%	13%	16%	19%	5%
	node 89	14%	24%	21%	27%	10%	4%
Turb.	node 09	12%	20%	4%	33%	27%	4%
	node 89	9%	18%	5%	46%	15%	6%
